# Blood parasites (*Trypanosoma*, *Leucocytozoon*, *Haemoproteus*) in the Eurasian sparrowhawk (*Accipiter nisus*): diversity, incidence and persistence of infection at the individual level

**DOI:** 10.1186/s13071-022-05623-x

**Published:** 2023-01-14

**Authors:** Milena Svobodová, Ivan Čepička, Lenka Zídková, Aysheshm Kassahun, Jan Votýpka, Lubomír Peške, Kristýna Hrazdilová, Jana Brzoňová, Petr Voříšek, Karel Weidinger

**Affiliations:** 1 Department of Parasitology, Faculty of Science, Charles University, Prague, Czechia; 2 Department of Zoology, Faculty of Science, Charles University, Prague, Czechia; 3 Prague, Czechia; 4 Department of Chemistry and Biochemistry, Mendel University, Brno, Czechia; 5 Biomedical Center, Faculty of Medicine in Pilsen, Charles University, Plzeň, Czechia; 6 Czech Society for Ornithology, Prague, Czechia; 7 Department of Zoology, Faculty of Science, Palacký University, Olomouc, Olomouc, Czechia

**Keywords:** Avian blood parasite, Haemosporida, Raptor, Birds of prey, Parasite persistence, *Trypanosoma avium*, *Trypanosoma corvi*, *Trypanosoma bennetti*

## Abstract

**Background:**

A high prevalence of parasites may result from life-long persistence of infection or from high reinfection rates. We have studied blood parasites in a breeding population of the accipitrid raptor, Eurasian sparrowhawk (*Accipiter nisus*), to determine parasite diversity and turnover.

**Methods:**

During this 7-year study, 210 adult Eurasian sparrowhawks breeding in the city of Prague were checked for parasites using several diagnostic methods.

**Results:**

In both female and male raptors, parasites of the genus* Leucocytozoon* were the most prevalent (92% and 85%, respectively) followed in decreasing order of prevalence by those of genus* Trypanosoma* (74% and 68%, respectively) and genus* Haemoproteus* (46% and 16%, respectively). The prevalence of all parasites increased with age in both sexes, with the females at each respective age having the higher prevalence. There was a positive association between *Haemoproteus* and *Leucocytozoon* infections. Persistence at the individual level was higher than incidence for *Trypanosoma* and *Haemoproteus*. In the case of *Leucocytozoon* and *Trypanosoma*, most individuals probably become infected in their first year of life or even before dispersal from the nest. The detected parasites belonged to *Trypanosoma avium* sensu stricto, *Leucocytozoon* sp. (haplotypes ACNI1 and ACNI3) and *Leucocytozoon mathisi* (haplotype ACNI4) and two new lineages of the *Haemoproteus elani* complex (ACCNIS6 and ACCNIS7). Detailed analysis of parasite lineages in individuals that were repeatedly sampled revealed lineage turnover that would otherwise remain hidden. Phylogenetic analysis revealed that the detected *Haemoproteus* belongs to a phylogenetically distant group whose taxonomic position requires further analysis.

**Conclusions:**

All three genera of blood parasites persist in infected individuals, thus enabling sustainability of vector transmission cycles. Prevalence increases with age; however, there is a high turnover of *Leucocytozoon* lineages. No clear evidence of parasite-induced mortality was found, and most of the individuals were infected early in life, particularly in the case of *Leucocytozoon*.

**Graphical abstract:**

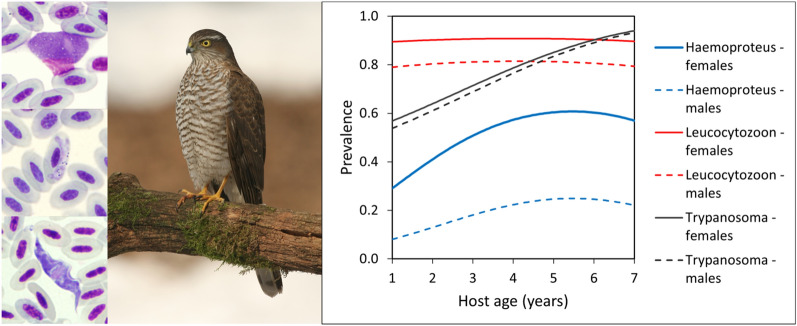

**Supplementary Information:**

The online version contains supplementary material available at 10.1186/s13071-022-05623-x.

## Background

Avian blood protists are widespread heteroxenous parasites which belong to two different supergroups, namely Discoba (trypanosomes, Kinetoplastea) and Alveolata (haemosporidians, Haemospororida) [1], with both groups transmitted by bloodsucking dipteran vectors. These parasites, especially haemosporidians, have regained attention in recent years due to the increasing application of molecular barcoding [2]. Despite new information on their prevalence and diversity, little is known about infection dynamics at the individual level. Moreover, researchers studying haemosporidians usually focus on members of the genus *Plasmodium* and genus *Haemoproteus* while data on blood parasites belonging to genus *Leucocytozoon* are relatively scarce (e.g. [3]) and, with few exceptions, trypanosomes remain largely neglected [4–6]. From the host point of view, due to the relative ease of capture and handling, passerines are the most studied hosts, with studies on raptors usually focusing on injured/captive individuals or nestlings [7, 8].

The Eurasian sparrowhawk (*Accipiter nisus*) (referred to hereafter as the sparrowhawk) is a small- to middle-sized raptor breeding nearly across the whole of Europe [9]. The species feeds almost exclusively on small birds, mainly passerines. The female, the larger and heavier sex, cares for young chicks in the nest, particularly in the first 2 weeks after hatching. The male is responsible for food provision during nest care, although the female also takes part in this role as the chicks become older. The sparrowhawk builds open nests in trees, the clutch is incubated for approximately 35 days and chicks spend 4–5 weeks at the nest plus 1–2 weeks in its surroundings. By 24–28 days after hatching, the young birds start to perch on branches near the nest and take their first flight. They are fed by their parents for a further 28–30 days and stay close to the nest while growing and practicing flying. Czech populations of sparrowhawk comprise vagrants or short-distance migrants to southwestern Europe [10, 11].

Blood parasites of the genera *Haemoproteus* and *Leucocytozoon* have been reported from sparrowhawks using microscopy [12, 13] and also molecular methods [8, 14–17]. In addition to haemosporidian parasites, the sparrowhawk also hosts trypanosomes belonging to the *Trypanosoma avium* and *Trypanosoma bennetti* groups [18, 19].

In a previous study, we compared the overall prevalence of blood parasites of the genera *Trypanosoma*, *Leucocytozoon* and *Haemoproteus* in nestlings and adults of the Common Buzzard (*Buteo buteo*) and sparrowhawk and found that older nestlings, as well as those sampled later in the season, had a higher probability of infection [20]. In the study reported here, we investigated the molecular diversity and persistence of *Leucocytozoon*, *Haemoproteus* and *Trypanosoma* parasites at the individual level in adult birds of both sexes in a long-term study of a sparrowhawk population. Our knowledge of the diversity and persistence of raptor blood parasites is incomplete due to the reasons described above despite the possibility that parasite persistence in infected hosts may be critical for parasite sustainability in host populations. Vertebrate hosts, on average, live longer than vectors; thus, in temperate regions, parasites must persist in vertebrates during the non-transmission season while vectors are unavailable since most vectors overwinter as immature life stages or before taking a blood meal. We hypothesized that due to the restricted seasonal availability of vectors, persistence of blood parasites in vertebrate hosts would be necessary for transmission to occur in the following season. Long-term persistence would then lead to an increasing prevalence of blood parasites in older individuals if the parasite is not so pathogenic that a significant proportion of infected individuals were to be eliminated from the population.

## Methods

### Study area and sampling

Blood parasites were studied in an urban breeding population of the sparrowhawk in the city of Prague, Czechia (49.99–50.14 N, 14.30–14.62 E), as described elsewhere [20]. The total study area is about 500 km^2^ and covers the area of the city and its suburbs. Habitats include built-up areas, parks, gardens and orchards as well as small tracts of woodland. Sparrowhawks breed in trees, usually in parks and gardens, across the whole area, including the city center. The population size has been estimated to be between 90 and 120 breeding pairs [21]. The population was sampled for parasites during the breeding seasons 1996–2002 (mid-June to July). Adults were captured close to their active nests using mist nets and a stuffed Eagle Owl (*Bubo bubo*) as a decoy. The birds were aged according to Newton and Hardey et al. [11, 22], or based on ringing dates of nestlings in the case of recaptures. For the purpose of this study, age 1 refers to an individual hatched in the previous year. Blood samples (125 µl) were taken from the brachial vein using a heparinized tuberculin syringe, and blood smears were prepared immediately, air dried, fixed with absolute methanol and stained with Giemsa (Sigma-Aldrich, St. Louis, MO, USA). A portion of the collected blood was used for trypanosome cultivation as previously described [18], and the remaining blood was fixed in 96% ethanol and stored frozen for subsequent DNA extraction. In the case of culture contamination, samples were excluded from the analysis; resulting trypanosome isolates were stored in liquid nitrogen for further use.

### Microscopy

Microscopic examination of blood smears was performed at 1000× magnification for 10 min, which in the present study corresponded to approximately 30,000 erythrocytes. Slides were also checked at 160× magnification for 5 min to look for low-density *Leucocytozoon* infections. Microphotographs of the parasites were taken at 1000× magnification with a CDC camera (DP70) on an Olympus BX51 microscope (Olympus Corp., Tokyo, Japan).

### DNA extraction, PCR amplification and sequencing

For the molecular study, the stored blood samples were first placed in a thermoshaker at 37 °C to evaporate the ethanol. DNA was then extracted using the High Pure PCR Template Preparation Kit (Roche Applied Science, Penzburg, Germany) according to the manufacturer’s instructions. The specific PCR protocol for each genus of parasite is described in detail in the following three subsections. Negative controls were used for all PCR assays, with one negative control (master mix with primers and PCR water instead of template DNA) included per 10 tested samples. The products of the PCR assays were analyzed by electrophoresis in 1% agarose gels, stained with SYBR Safe DNA gel stain and visualized under UV light. Positive PCR products were directly purified using ExoSAP-IT™ (Thermo Fisher Scientific) or were cut from the gel in the case of double bands and purified using the High Pure PCR Product Purification Kit (Roche Applied Science) according to the manufacturer’s instructions and sent for sequencing to the Core facility of the Faculty of Science, Charles University.

#### *Leucocytozoon*

A cytochrome* b* gene fragment was amplified using a nested PCR method [23]. The first amplification round consisted of 35 cycles performed in a final volume of 16 μl of PCR mix (EmeraldAmp MAX HS PCR Master Mix; TaKaRa Bio Inc., Kusatsu, Shiga, Japan) with primers DW2 (5ʹ-TAATGCCTAGACGTATTCCTGATTATCCAG-3ʹ) and DW4 (5ʹ-TGTTTGCTTGGGAGCTGTAATCATAATGTG-3ʹ) and an annealing temperature of 60 °C for 20 s. The next step in the nested PCR consisted of 35 amplification cycles; 1 μl of the amplified product was used as a template with primers DW1 (5ʹ-TCAACAATGACTTTATTTGG-3ʹ) and DW6 (5ʹ-GGGAGCTGTAATCATAATGTG-3ʹ) and an annealing temperature of 50 °C for 20 s. DNA from passerine blood was used as the positive control.

#### *Haemoproteus*

In order to detect possible *Leucocytozoon* and *Haemoproteus* co-infections, we designed a nested PCR assay. For the first round, primers HaemNFI (5ʹ-CATATATTAAGAGAAITATGGAG-3ʹ) and HaemNR3-modif (5ʹ-ATARAAAGGTARGAAATACCATTC-3ʹ) targeting the 5ʹ end of the mitochondrial cytochrome* b* gene from *Leucocytozoon*, *Haemoproteus*, and *Plasmodium* were adopted from Hellgren et al. [24]. The reverse primer was slightly modified (2 bases were degenerated) to take account of the diversity of the growing number of available sequences. For the second round of PCR, we designed a highly specific pair of primers that amplify *Haemoproteus* exclusively. The forward primer HaemF-modif (5ʹ-ATGGTGTTTTAGATATATGCATG-3ʹ), which amplifies *Haemoproteus* and *Plasmodium* targets, was adopted from Bensch et al. [25] and was modified following the alignment of available *Haemoproteus* sequences. The reverse primer HaemRspec (5ʹ-GTAATGGAGTCACAAATARACTAAC-3ʹ) was newly designed based on the alignment of available *Haemoproteus* sequences originating from the birds of prey available in GenBank and allowed exclusive amplification of this genus. This nested PCR was performed with blood samples from repeatedly sampled individuals. The first round of the nested PCR (PrimeStar Master Mix; TaKaRa Bio Inc.) was performed in a final volume of 16 μl at the following thermocycling parameters: 3 min of denaturation at 98 °C; followed by 35 cycles of 98 °C for 10 s, 46 °C for 30 s, and 35 s for 72 °C; with a final elongation step at 72 °C for 10 min. The second round of nested PCR was performed in a final volume of 24 μl was done under the same conditions as for the first round, but with an annealing temperature of 48 °C. Given the specificity of the protocol, DNA from sparrowhawk blood positive for *Haemoproteus* was used as the positive control.

#### *Trypanosoma*

For lineage identification, frozen cultures were thawed and passed on blood agar in flat tubes. Cells from log-phase cultures were washed 3 times in normal saline and used for DNA isolation. For lineage identification, the trypanosome 18S rRNA gene was amplified as described previously [19]. For sequencing, the internal primer SSU-1000-R (ATGCCTTCGCTGTAGTTCGTCT; own design) was used. Sequences obtained were identified using the BLASTn algorithm (https://blast.ncbi.nlm.nih.gov/Blast.cgi). All sequences unambigously matched one of the lineages of avian trypanosomes in GenBank with 100% identity.

### Phylogeny

For initial assessment of the phylogenetic position for newly determined sequences (ACNI1, ACNI03, ACNI04, ACCNIS6, ACCNIS07), we downloaded the entire MalAvi database (http://130.235.244.92/Malavi/; 24 April 2022) and added unique variants of the newly determined sequences. The sequences were aligned using the MAFFT method [26] on the MAFFT 7 server (https://mafft.cbrc.jp/alignment/server/) with the G-INS-i algorithm. The alignment was manually edited in BioEdit 7.0.4.1 [27]. The final masked data set contained 478 positions. Phylogenetic trees were constructed by the maximum likelihood method, using RAxML 8.0.0. [28] under the GTRGAMMAI model, with 100 starting trees. Bootstrap support values were generated in RAxML from 1000 pseudoreplicate data sets. Full-length sequences identified as possible close relatives of our newly determined sequences, sequences representing a broad sampling of haemosporidians and available sequences of *Haemoproteus* and *Leucocytozoon* from raptors were retrieved from GenBank. These, together with our newly determined sequences, were used to construct the final data set. The phylogenetic analysis was carried out as described above (the final data set consisted of 1004 aligned characters).

### Statistical analysis

Raw prevalence was calculated for each parasite and parasite combination as the proportion of positive host individuals categorized by age and sex. Multiple samples from the same host individuals were treated as independent observations (see below for a mixed-model approach). The effects of multiple predictors on the infection status of host individuals (0/1) were evaluated by fitting separate logistic regressions for each parasite genus. A model with either linear or quadratic effect of host age (fixed-effect continuous predictor) was run for a data set with and without the two oldest female hosts (aged 9 years), resulting in four models for each parasite genus. Age was centered before analysis, so that both linear and quadratic terms could be interpreted from the quadratic model. All models included host sex and year (6 levels) as fixed-effect categorical predictors. Although the effect of year was not of primary interest and was not statistically significant in any model, it was kept as a blocking variable to account for annual variation. An effect of host age and sex on the prevalence of multiple infections (coded as: negative, single, double, triple) was evaluated by fitting with a multinomial logistic regression. An effect of year was not included since the data set was too sparse and the models did not converge.

The available data set represents a mixture of cross-sectional (across individuals) and longitudinal (within individuals) data as some individuals were sampled repeatedly (2–5 times), resulting in a total of 253 samples from 190 individuals for *Haemoproteus*, 256 samples from 193 individuals for *Leucocytozoon* and 209 samples from 154 individuals for *Trypanosoma* (see Results section). Hence it would be correct to account for non-independence among multiple samples from the same individual. However, the mixed logistic model that contained individual identity as a random effect did not converge for *Leucocytozoon* and *Trypanosoma*. As this was an exploratory study, in which we focused on detecting patterns in the data rather than on formal hypothesis testing, we preferred the same modeling approach applied to all parasite genera. To check for an effect of pseudoreplication on parameter estimates and statistical inference, we reran all models for all parasite genera with a data set that contained only one (the first) sample for each individual. Because the results were consistent in both qualitative and quantitative terms (Additional file 1: Tables S1, S2) and because for all parasite genera the degree of pseudoreplication was not high (76–79% individuals sampled only once) and infection status was reversible, we hereafter present results based on the complete data set analyzed as independent data.

Associations among parasite genera was evaluated in two ways. To allow for direct comparison with our previous results [20], we first replicated the analysis with an updated data set. We constructed a 2 × 2 × 2 (*Trypanosoma* × *Leucocytozoon* × *Haemoproteus*) contingency table from pooled data and fitted a Poisson regression to the eight cell frequencies with an added constant of 0.5. We first fitted the full model to check for a three-way interaction (interpreted as triple associations among parasite genera). Next, we removed the three-way interaction and examined the reduced model with all two-way interactions (interpreted as double associations between parasite genera). Then, we evaluated double associations among parasite genera while accounting for simultaneous effects of age, sex, and year. This was done by including infection status for one parasite genus as an additional fixed-effect predictor in the logistic regression where infection status of the second parasite genus was the response variable, and vice versa [29].

To analyze within-individual changes of infection status (0/1) in repeatedly sampled host individuals we counted frequencies of the four possible scenarios: gain of infection (0-1), loss of infection (1-0), retention of negative status (0-0) and retention of positive status (1-1). Next, we ran logistic regression models that included the infection status on the first sampling (together with age and sex) as a fixed-effect predictor and the infection status on subsequent sampling as a response. This is equivalent to testing independence in a 2 × 2 contingency table, but the present models account for covariates. Estimated prevalence of originally uninfected individuals indicates incidence, while estimated prevalence of originally infected individuals indicates persistence. Finally, we ran separate logistic regressions for incidence and prevalence that included age and sex as fixed-effect predictors. A data unit in all the above analyses was one interval (≥ 1 year) between repeated sampling of the same individual; individuals sampled more than twice (21–28% of those sampled repeatedly) thus contributed more than one interval (see Results section). We did not apply a mixed model approach to account for non-independence for the same reasons as in analyses of age specific prevalence (see explanation above).

The available sample sizes for parasite lineages were insufficient for modeling, so only raw frequencies or counts are presented. Dependence of infection status for *Leucocytozoon* lineages between two subsequent samplings of the same individual was evaluated by Fisher’s exact test.

Raw and model-estimated frequencies are presented with the binomial 95% confidence interval (CI). Effect sizes are presented as odds ratios (OR) derived from estimates of logistic regression (OR = e^Estimate^). Inference on parameter estimates is based on 95% CI and approximate *P*-values are based on *Z*-scores. Inference on interactions (and main effects with more parameters) is based on a likelihood-ratio test (LRT) comparison of models with and without the particular term. All analyses were conducted using packages “lme4ˮ and “nnetˮ within the program R [30].

## Results

### Sampled individuals and detected parasite genera

**Fig. 1:**
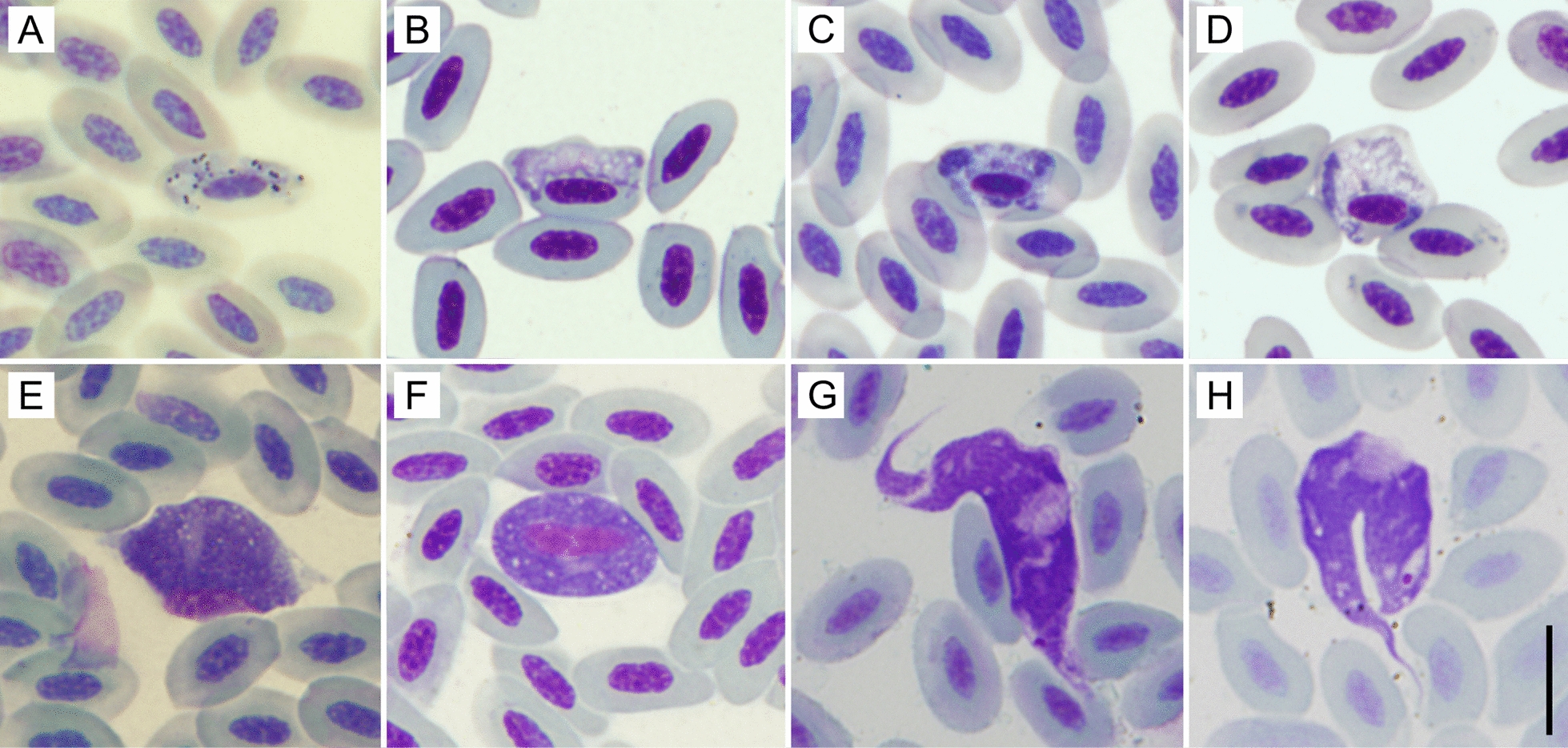
Giemsa-stained blood smears from sparrowhawks. **a**–**d** *Haemoproteus elani* complex. **a** A gametocyte with hemozoin granules, **b**–**d** gametocytes missing hemozoin granules: **a**,** b** *Haemoproteus* lineage ACCNIS06, **c**,** d** *Haemoproteus* lineage and ACCNIS07. **e** *Leucocytozoon* sp., **f** *Leucocytozoon mathisi*. **g**, **h** trypomastigotes of *Trypanosoma avium* sensu stricto. Scale bar: 10 µm (**a**–**h**)

A total of 273 samples were collected from 210 adult sparrowhawks (117 females, 93 males). The exact age of the host could be determined for 258 of the obtained samples. Forty-six individuals were repeatedly sampled (33 were tested twice; 11 were tested 3 times; 2 were tested 5 times) in different breeding seasons. Parasites belonging to the genera *Haemoproteus*, *Leucocytozoon* and *Trypanosoma* were detected (see Fig. 1). *Plasmodium* and microfilariae were not found.

### Parasite prevalence

The overall prevalence of each individual parasite genus, as well as prevalence according to age and sex, is given in Table 1. Blood smears were not available for some samples; in these cases, the infection status of haemosporidians was determined by PCR only (14 samples tested for *Haemoproteus*, 17 for *Leucocytozoon*). Our newly developed specific primers detected *Haemoproteus* infection in 44 out of 88 blood samples from repeatedly tested individuals; 28 sequences of adequate quality were obtained.

**Table 1:** The prevalence of *Haemoproteus*, *Leucocytozoon* and *Trypanosoma* infections in adult sparrowhawks, categorized by host sex and age

Age (years)	Females									Males							
	1	2	3	4	5	6	7	9	Pooled	1	2	3	4	5	6	7	Pooled
Sample size																	
n (H)	40	36	32	13	16	9	9	2	157	32	29	21	10	1	2	1	96
n (L)	40	37	32	13	16	9	9	2	158	33	30	21	10	1	2	1	98
n (T)	31	31	29	9	11	9	6	2	128	29	21	19	8	1	2	1	81
n (HLT)	31	30	29	9	11	9	6	2	127	28	21	19	8	1	2	1	80
Overal prevalence (%)																	
H	32.5	52.8	43.8	53.8	50.0	77.8	55.6	0.0	46.5	3.1	10.3	33.3	30.0	100.0	0.0	0.0	15.6
L	87.5	91.9	100.0	92.3	87.5	100.0	88.9	50.0	91.8	81.8	96.7	76.2	76.0	100.0	100.0	100.0	84.7
T	54.8	74.2	82.8	77.8	81.8	88.9	100.0	50.0	74.2	58.6	76.2	63.2	75.0	100.0	100.0	100.0	67.9
No infection (%)																	
0	6.5	0.0	0.0	0.0	0.0	0.0	0.0	0.0	1.6	17.9	0.0	10.5	0.0	0.0	0.0	0.0	8.8
Single infection (%)																	
H	0.0	0.0	0.0	0.0	0.0	0.0	0.0	0.0	0.0	0.0	0.0	0.0	0.0	0.0	0.0	0.0	0.0
L	19.4	16.7	13.8	11.1	9.1	0.0	0.0	50.0	14.2	21.4	23.8	21.1	25.0	0.0	0.0	0.0	21.3
T	6.5	3.3	0.0	0.0	9.1	0.0	16.7	50.0	4.7	3.6	4.8	15.8	25.0	0.0	0.0	0.0	8.8
Double infection (%)																	
HL	19.4	6.7	3.4	11.1	9.1	11.1	0.0	0.0	9.4	0.0	0.0	5.3	0.0	0.0	0.0	0.0	1.3
HT	0.0	0.0	0.0	0.0	0.0	0.0	0.0	0.0	0.0	0.0	0.0	0.0	12.5	0.0	0.0	0.0	1.3
LT	35.5	16.7	37.9	33.3	27.3	22.2	16.7	0.0	28.3	53.6	66.7	26.3	12.5	0.0	100.0	100.0	47.5
Triple infection (%)																	
HLT	12.9	56.7	44.8	44.4	45.5	66.7	66.7	0.0	41.7	3.6	4.8	21.1	25.0	100.0	0.0	0.0	11.3

* H* *Haemoproteus*,* L* *Leucocytozoon*,* T* *Trypanosoma*

For both females and males, blood parasites of genus *Leucocytozoon* were the most prevalent (92% and 85%, respectively) followed in decreasing order of prevalence by those of genus *Trypanosoma* (74% and 68%, respectively) and genus *Haemoproteus* (46% and 16%, respectively). Overall prevalence of blood parasites was consistently higher in females, with the largest between-sex difference found for *Haemoproteus*. Only 2% of females and 9% of males were negative for any blood parasite.

### Parasite species and their phylogeny

**Fig. 2:**
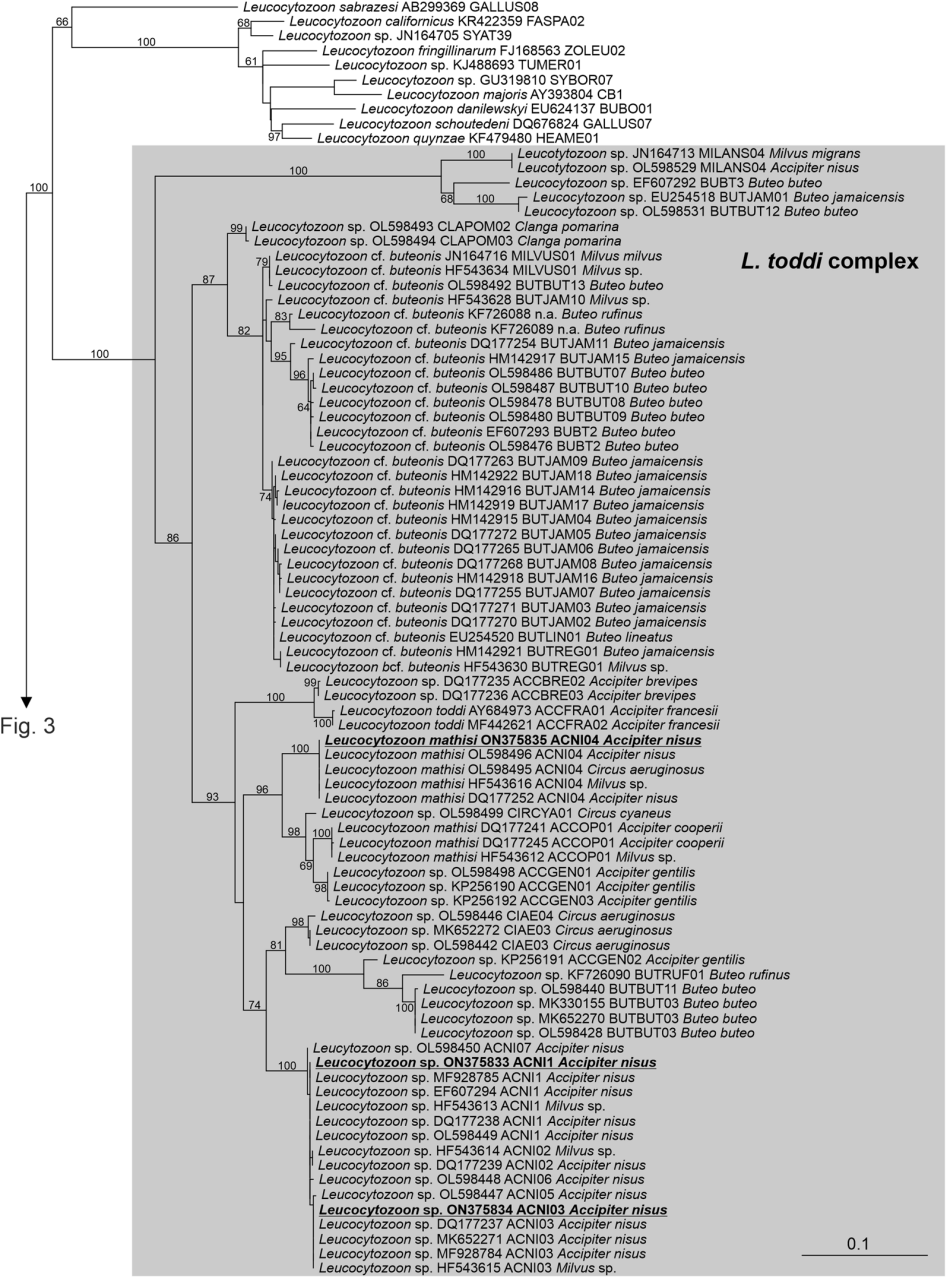
A portion of the phylogenetic tree of haemosporidia showing the subtree of *Leucocytozoon*. The values at branches represent statistical support in bootstrap values (RAxML); bootstrap values below 50 are not shown. New sequences are shown in bold and underlined. The haplotypes of sequences obtained from birds are shown. Host names are shown within the *Leucocytozoon toddi* complex

**Fig. 3:**
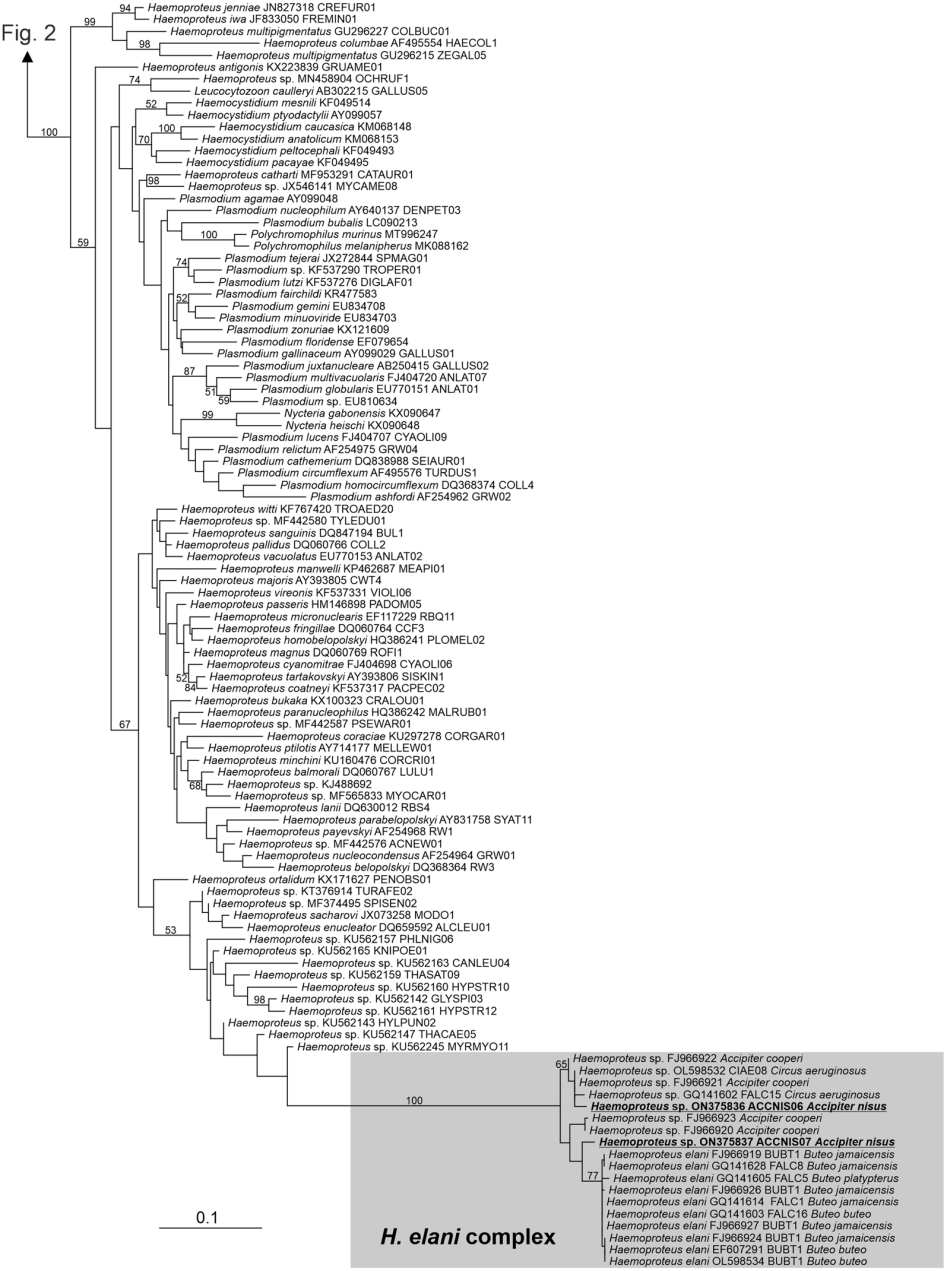
A portion of the phylogenetic tree of haemosporidia showing the subtree of *Haemoproteus*, *Haemocystidium*, *Plasmodium*, *Nycteria* and *Polychromophilus*. The values at branches represent statistical support in bootstrap values (RAxML); bootstrap values below 50 are not shown. New sequences are shown in bold and underlined. The haplotypes of sequences obtained from birds are shown. Host names are shown within the *Haemoproteus elani* complex

The phylogenetic tree of haemosporidian parasites inferred from the cytochrome* b* gene is shown in Figs. 2 and 3. The genus *Leucocytozoon* was recovered as monophyletic with maximum support (Fig. 2). All our *Leucocytozoon* sequences branched within a robustly supported clade composed exclusively of sequences obtained from the blood of accipitrid raptors, here referred to as the *Leucocytozoon toddi* complex. These sequences represented three genetic lineages, whose barcode region corresponded to haplotypes ACNI1, ACNI03 and ACNI04, respectively. Species identity of the first two haplotypes is uncertain, while ACNI04 is considered to be *Leucocytozoon mathisi* [31].

*Haemoproteus* appeared paraphyletic, having the other genera as internal branches, but support of the topology was low (bootstrap values ≤ 59). All our *Haemoproteus* sequences branched within a robustly supported clade composed exclusively of sequences obtained from blood of accipitrid raptors, here referred to as the *Haemoproteus elani* complex (Fig. 3). This clade comprised several previously recognized haplotypes of *Haemoproteus* (BUBT1, CIAE08, FALC1, FALC5, FALC8, FALC15 and FALC16) as well as several haplotypes not included in the MalAvi database [32]. The only formally described species known to belong to the *H. elani* complex is *H. elani* de Mello, 1937 (Fig. 1); this species has highly pleomorphic gametocytes[33] and the only notable difference is the absence of hemozoin granules in some of the detected gametocytes (Fig. 1; 5 out of 24 gametocytes [21%]), and approximately a quarter of gametocytes are amoeboid. The *H. elani* complex produced a rather long branch in our tree with an uncertain phylogenetic position. Our sequences represented two variants, whose barcode region did not correspond to any known *Haemoproteus* haplotype. Our new lineages were designated MalAvi haplotypes ACCNIS06 and ACCNIS07.

Sixty trypanosome isolates were obtained by culture. With the exception of four isolates, all isolates (93.3%) belonged to *Trypanosoma avium* sensu stricto (*T. avium* s.s.; lineage 10 + 11). The four exceptions included three (5%) isolates belonging to *Trypanosoma corvi* (lineage 4) and one isolate (1.7%) belonging to lineage 8 of (*Trypanosoma bennetti* sensu lato [*T. avium *s.l.]) (for lineage details and trypanosome phylogeny see [19]). These rare isolates did not originate from the individuals that were repeatedly sampled.

### Host age and parasite prevalence

**Fig. 4:**
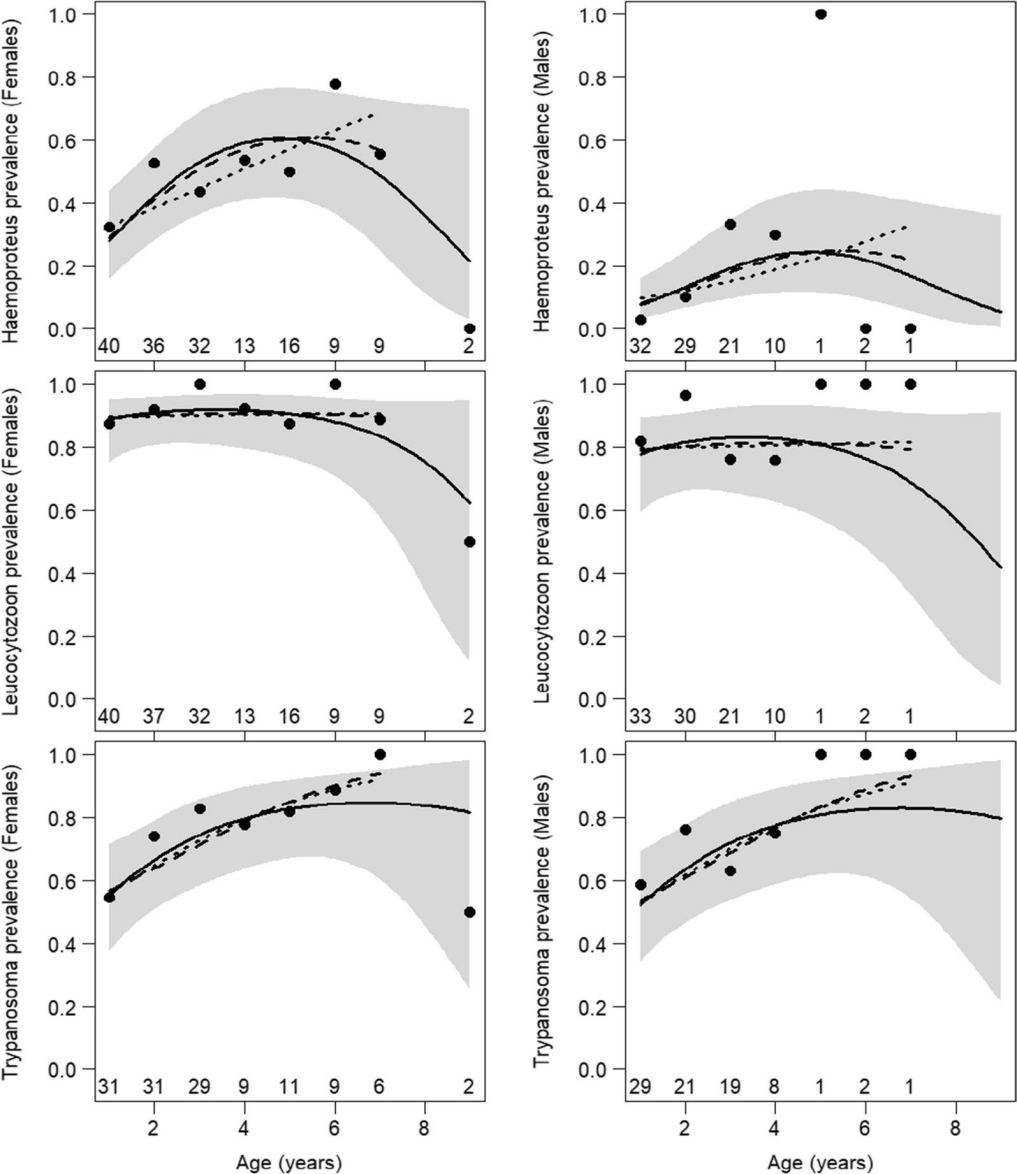
Effects of sparrowhawk age and sex on total prevalence of *Haemoproteus*, *Leucocytozoon* and *Trypanosoma* estimated by logistic model with additive effects of age, sex and year. Age was modeled as a quadratic effect for ages 1–9 years (solid line with shaded area showing the 95% confidence interval), as a quadratic effect for ages 1–7 years (dashed line) or as a linear effect for ages 1–7 years (dotted line). See Additional file 1: Table S1 for parameter estimates. Sample size (numbers along the age axis) and raw prevalence (points) calculated as a simple proportion of positive cases (see Table 1) are shown for each age category

Age-related patterns of prevalence were influenced by the range of analyzed data. Sufficient samples of inspected individuals were available for up to 7 years (females) and 4 years (males) of age (Table 1). Within this range, the prevalence of *Haemoproteus* and of *Trypanosoma* monotonically increased, while that of *Leucocytozoon* remained high and stable (Fig. 4; Additional file 1: Tables S1, S2). The oldest birds were represented by two 9-year-old females, of which one was infected by *Leucocytozoon*, the other by *Trypanosoma* and neither by *Haemoproteus* (Table 1). There was marginal statistical evidence for decreasing *Haemoproteus* prevalence in the oldest individuals only if these two birds were included (quadratic effect of age, OR = 0.91; 95% CI 0.84–0.98; *P* = 0.014), while conclusions for the prevalence of *Leucocytozoon* and *Trypanosoma* remained unchanged (Additional file 1: Tables S1, S2; Fig. 4). The above-mentioned effect of age was independent of sex; all models with age × sex interaction fitted worse than corresponding main effect models (LRT, all *P* > 0.06). The raw prevalence for each of the three parasite genera was higher in females than in males (Table 1). After accounting for age, prevalence for each of the three parasite genera in females was still higher than in males, but this effect was pronounced and statistically supported only for *Haemoproteus* (*P* < 0.001 in all examined models; Additional file 1: Tables S1, S2).

### Host age and coinfections with different parasite genera

**Fig. 5:**
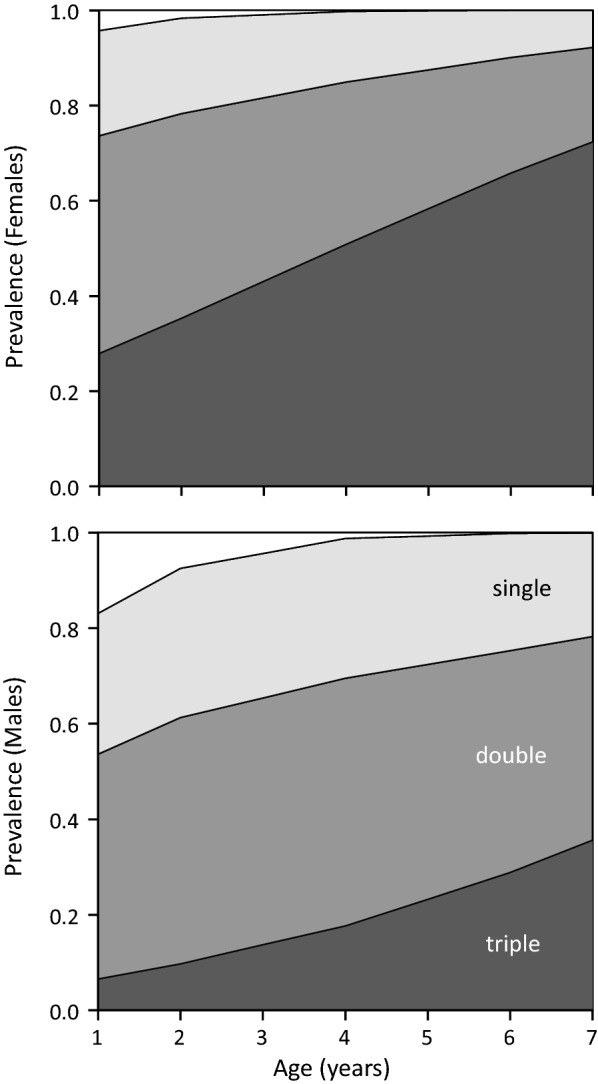
Effects of sparrowhawk age and sex on the prevalence of multiple infections by *Haemoproteus*, *Leucocytozoon* and *Trypanosoma*. Estimates of multinomial logistic model with additive effects of age and sex are shown. Increasing degree of shading indicates none, single, double and triple infections. Data on raw prevalence are given in Table 1

Coinfections were common across all age classes (Table 1). The relative prevalence of triple, double, single and no infections (multinomial response) varied with age (LRT, *χ*^2^ = 16.6, *df* = 3; *P* < 0.001) and sex (LRT, *χ*^2^ = 22.4, *df* = 3; *P* < 0.001), with no indication of age × sex interaction (LRT, *χ*^2^ = 2.7, *df* = 3; *P* = 0.443). The prevalence of triple infections increased with age at the expense of double and single infections, and the overall prevalence was higher in females (Fig. 5).

### Associations among parasites

Most females were infected by all three parasites (42%), followed by *Leucocytozoon/Trypanosoma* double infection (28%), while in males the opposite was true (47% *Leucocytozoon/Trypanosoma*, 11% *Haemoproteus/Leucocytozoon/Trypanosoma*). The most frequent single infection was by *Leucocytozoon* (14% in females, 21% in males) while *Haemoproteus* was never found as a single infection.

An analysis of the three-way contingency table of pooled data revealed a positive *Haemoproteus* ×* Leucocytozoon* association (LRT, *χ*^2^ = 11.6, *df* = 1; *P* < 0.001) and indicated a positive *Haemoproteus* ×* Trypanosoma* association (LRT, *χ*^2^ = 4.06, *df* = 1; *P* = 0.044); there was little evidence for a *Leucocytozoon* ×* Trypanosoma* association (LRT, *χ*^2^ = 1.55, *df* = 1; *P* = 0.213) or triple association among parasite genera (LRT, *χ*^2^ = 0.003, *df* = 1; *P* > 0.9). Further analyses of double associations accounted for simultaneous effects of age, sex and year on prevalence. In this case, the prevalence of *Haemoproteus* was higher for hosts infected with *Leucocytozoon* (OR = 3.76, 95% CI 1.16–17.10; *P* = 0.047) and vice versa (OR = 3.79, 95% CI 1.20–16.86, *P* = 0.041). The other two double associations were also positive, but without statistical support: *Haemoproteus* ×* Trypanosoma* (OR = 2.04, 95% CI 0.94–4.59; *P* = 0.077) and vice versa (OR = 2.03, 95% CI 0.94–4.58; *P* = 0.071), *Leucocytozoon* ×* Trypanosoma* (OR = 1.66, 95% CI 0.60–4.50; *P* = 0.320) and vice versa (OR = 1.58, 95% CI 0.57–4.32; *P* = 0.460). An effect of infection status by one parasite as a predictor of prevalence for another parasite was independent of age or sex (no evidence for interaction effects: LRT, all *P* > 0.09).

### Within-individual changes of infection status

Infection status on first sampling, after accounting for an effect of age and sex, influenced prevalence on subsequent sampling of the same individual for *Trypanosoma* (OR = 59, 95% CI 7–1346; *P* < 0.001) and *Haemoproteus* (OR = 7.10, 95% CI 2.27–24.40; *P* = 0.001) (Additional file 3: Figure S1). This means that persistence was higher than incidence for *Trypanosoma* and *Haemoproteus*. For *Leucocytozoon*, all previously uninfected individuals had gained infection at the next sampling (Table 2), so the incidence was 100%. No evidence was found for an effect of age and sex on either incidence or persistence analyzed separately in any parasite (all *P* > 0.1), but the sample size was small for these analyses (Table 2).

**Table 2:** Infection status for three parasite genera on consecutive repeated sampling of the same host individuals

Infection status	*Haemoproteus*			*Leucocytozoon*			*Trypanosoma*		
	Female	Male	Pooled	Female	Male	pooled	Female	Male	Pooled
0-0	14	10	24	0	0	0	6	2	8
0-1	9	0	9	8	2	10	3	2	5
1-0	7	0	7	1	2	3	1	0	1
1-1	21	2	23	42	8	50	26	4	30
*n (intervals)*	51	12	63	51	12	63	36	8	44
*n (individuals)*	34	12	46	34	12	46	26	8	34

Given is the number of cases (intervals) for each of the four possible scenarios: gain of infection (0-1), loss of infection (1-0), retention of negative status (0-0), retention of positive status (1-1)

Sample size indicates number of individuals and number of time intervals (≥ 1 year) between samplings; individuals sampled more than twice contributed more than one interval

Estimates of incidence and persistence are shown in Additional file 3: Figure S1

### *Leucocytozoon *and *Haemoproteus* lineages, and their persistence

*Leucocytozoon* sp. (ACNI1 and ACNI03) was far more common than *L. mathisi* (ACNI04) and, within *L.* sp., lineage ACNI1 was more prevalent than ACNI03 (Additional file 4: Figure S2). Infection status on first sampling (not accounting for covariates due to small sample size) influenced prevalence on subsequent sampling of the same individual for *Leucocytozoon* sp. (OR = 7.66; Fisher´s exact test, *P* = 0.030); a similar effect for *L. mathisi* was not statistically supported (OR = 3.21; Fisher´s exact test, *P* = 0.224) (Additional file 5: Figure S3). This means that persistence was higher than incidence for *Leucocytozoon* sp., but no clear pattern could be detected for *L. mathisi* and lineages of *Leucocytozoon* sp. (small sample size; Table 3). Infection status on consecutive sampling suggested that interchange between *Leucocytozoon* sp. and *L. mathisi* is about equally frequent in both directions (i.e. transition matrix is approximately symmetrical; Table 4). On the other hand, lineage ACNI1 was never replaced by lineage ACNI03, but the reverse was found (Table 4).

**Table 3:** Infection status for *Leucocytozoon* species and haplotypes of *Leucocytozoon* sp. on consecutive repeated sampling of the same host individuals

Infection status	*Leucocytozoon* species		Haplotype of *Leucocytozoon* sp.	
	*Leucocytozoon* sp.	*Leucocytozoon mathisi*	ACNI1	ACNI03
0-0	4	20	0	6
0-1	4	6	3	9
1-0	3	5	0	4
1-1	25	5	18	2
n (Intervals)	36	36	21	21
n (Individuals)	29	29	16	16

Data represent a subset of the data shown in Table 2, for which the species and haplotype could be identified

Estimates of incidence and persistence are shown in Additional file 5: Figure S3

**Table 4:** Transition matrices of infection status between consecutive repeated sampling of the same host individuals

Original status	Subsequent status		
*Leucocytozoon* species	Leucocytozoon sp.	Mixed	Leucocytozoon mathisi
*Leucocytozoon* sp.	20	3	3
Mixed	2	0	0
L. mathisi	3	1	4
*Leucocytozoon* sp. haplotype	ACNI1	Mixed	ACNI03
ACNI1	6	9	0
Mixed	2	1	0
ACNI03	2	1	0
*Haemoproteus* haplotype	ACCNIS06	Mixed	ACCNIS07
ACCNIS06	4	0	0
Mixed	0	2	0
ACCNIS07	0	0	2

Given is the number of cases (intervals) for each of the nine possible scenarios

Data for *Leucocytozoon* species (*n* = 36) and haplotypes (*n* = 21) correspond to those shown in Table 3

Lineages of *Haemoproteus* remained unchanged between repeated sampling of the same individuals but only a small proportion of repeatedly sampled individuals were infected at both samplings (Table 4). Due to the low prevalence of *Haemoproteus*, the number of sequences obtained from individuals that repeatedly tested positive for *Haemoproteus* was too low to perform a formal statistical analysis of lineage persistence. *Haemoproteus* barcoding based on 28 sequences of adequate quality obtained from repeatedly sampled individuals revealed two distinct genotypes. Of these, 21 sequences belonged to a novel haplotype ACCNIS06 (sequence homology to the most similar already published haplotype, BUBT1, was 97.92%), while seven belonged to the haplotype ACCNIS07 (sequence homology to the most similar already published haplotype, CIAE08, was 99.15%). Four obtained sequences revealed mixed infections.

## Discussion

The mechanisms of parasite persistence in host populations are still poorly understood. In the present study, we studied blood parasites belonging to three genera in a breeding sparrowhawk population that has been followed in a long-term survey. Repeated sampling of adult individuals allowed us to follow persistence, incidence and age-related patterns of the infections.

The detected lineages of the *L. toddi* complex were previously reported from sparrowhawks; however, to our knowledge, we are the first to find lineages belonging to the *H. elani* complex in the sparrowhawk (for review see [8]). Phylogenetic analysis revealed that the detected *Haemoproteus*, together with lineages found previously and assigned morphologically by G. Valkiunas to the morphospecies *H. elani* de Mello, 1937 [32], might represent a distinct genus. Parasites belonging to this clade have previously been found in other accipitrid raptors. Krone et al. [15] assigned it to the genus *Plasmodium*, but its position was unsupported. Outlaw and Ricklefs [34] designated it as an “unknown genus,ˮ potentially being *Plasmodium*. The taxonomic position of parasites of the *H. elani* complex needs to be elucidated.

There are only few records of molecularly characterized trypanosomes infecting the genus *Accipiter*. In addition to strains originating from the sparrowhawk population studied here, *T. avium* s.s. was found in the Goshawk (*Accipiter gentilis*) and Japanese sparrowhawk (*Accipiter gularis*) [5, 19]. Barcoding of trypanosome isolates obtained in our study revealed that the vast majority of the birds harbored trypanosomes belonging to *T. avium* s.s. In buzzard populations sampled in Czechia, the spectrum of trypanosome lineages was similar, with only three out of 83 barcoded isolates belonging to *T. corvi*, while 96% belonged to *T. avium* s.s. (Svobodová and Kassahun, unpublished). We hypothesize that striking differences in the prevalence of different trypanosome lineages are not caused by raptor resistence to these lineages but rather by different exposure to vectors or by different transmission modes of the respective trypanosome species. Vectors differ in their height preferences, with black flies being found almost exclusively in the canopy level [35], thus facilitating transmission of *T. avium* to birds that perch or build their nests in the canopy. Moreover, all avian trypanosomes with life-cycles that have been elucidated to date are transmitted by vector ingestion; some of them may also use transconjuctival transmission via prediuresis of infectious stages (*T. avium* s.s., *Trypanosoma thomasbancrofti* [36, 37]). These species do not depend exclusively on vector ingestion and thus may enter potential hosts that are not willing to eat the infected vector.

The prevalence of trypanosomes based on their detection on blood smears is usually low in birds, including members of order Accipitriformes. Munoz et al. [38] did not find any infection in 22 sparrowhawks screened; however, Hanel et al. [39] found trypanosomes in nine out of 15 sampled goshawks. In our sample, we found trypomastigotes on 14 slides out of the 254 screened (5.5%), while using the culture method, 74% of adults were positive for trypanosomes [20]. Thus, cultivation was more sensitive than microscopy for trypanosome detection by an order of magnitude.

PCR diagnosis of raptor haemosporidians is also limited by a number of pitfalls. The most popular protocol used to detect haemosporidia has been developed for passerines, and there is evidence that this protocol is not optimal for the detection of raptor parasites [8, 24, 39]. In the present study, we used the DW primers designed by Perkins and Schall [23] for the detection of *Leucocytozoon* infections, but we developed a specific PCR protocol, including newly designed degenerate primers, for the detection of *Haemoproteus* infections.

Our previous study, based on blood culturing methods and blood smears, revealed a prevalence of 74% for *Trypanosoma*, 88% for *Leucocytozoon* and 30% for *Haemoproteus* [20]. The present study shows that sex of the sampled birds has a significant influence on the respective prevalence; the largest difference between males and females was the 31% pooled prevalence of *Haemoproteus*. This difference is substantial, and host sex should be taken into account in comparative studies in addition to host species and age.

In both females and males, the prevalence of trypanosomes and of *Haemoproteus* increased with age, although in females the increase was greater; *Leucocytozoon* prevalence remained high and stable in both sexes. A high prevalence of haemosporidians in the sparrowhawk has been found previously in Scotland, with 92% of adult females and 93% of adult males testing positive for *Leucocytozoon*; in that study the prevalence of *Haemoproteus* was lower and, similarly to our study, differed between females and males (32% vs 17%) [12]. Similar factors may drive prevalence patterns across different populations of the same species. On the contrary, no sex differences in the prevalence of *Haemoproteus* and *Leucocytozoon* infections were found in the Black Sparrowhawk (*A. melanoleucus*) in South Africa, but the exact age of the adults was not assessed in that study [40].

The higher prevalence of infections in females might result from increased exposure to parasite vectors at the nest and/or, in the case of the sparrowhawk, to a larger size, with females being larger than males, from a higher production of kairomones that attract vectors. The lower infection prevalence in males of the same age might also result from differences in ontogenetic development early in life. Males mature faster, become feathered earlier and leave nests 3–4 days earlier than their female counterparts (sisters) [41]. If a substantial part of the infections is acquired at the nest, these factors could also partly explain the higher prevalence of infections in females.

Ashford et al. [42] speculated that transmission of haemosporidia in a sparrowhawk population occurs almost exclusively during breeding, based on the observations that prevalence in adults is not higher than in nestlings and that besides breeding, the possibility of an infectious bite by an individual vector infected with a specific parasite is very low. This vector-mediated parent-to-offspring transmission was later confirmed in another common accipitrid species, the buzzard, and its *Leucocytozoon* parasite [43]. Parent-to-nestling transmission probably occurs in other avian apicomplexan parasites as well [44].

There is some evidence that blood parasites (*Haemoproteus*) cause selective avian mortality, leading to a lower prevalence in the older age classes [45]. In our case, the decrease in prevalence was only statistically significant for *Haemoproteus*, but only after including the two oldest individuals (two 9-year-old females) to the analysis. The maximal life span of a female sparrowhawk is around 10 years, and there is evidence for lower survival rates in the older age classes (7–10 years) [46]. It is possible that parasites influence survival in concordance with senescence, which leads to decreased immunocompetence [47, 48].

In our previous study, modeled *Leucocytozoon* prevalence at fledging was around 30% [20]. Since the prevalence in year-after-hatching adults exceeds 80%, most individuals must become infected with *Leucocytozoon* during their first year of life. This prepatent period (first nestling found positive for *Leucocytozoon* at the age of 17 days [20]) implies that the majority of the individuals are infected before postfledging dispersal. The predicted prevalence of trypanosomes at fledging was similar in both studies (present study and previous study [20]), but its increase was slower in our previous study, which again corresponds to lower trypanosome prevalence (56%) in the year-after-hatching birds in the present study.

We suggested previously that the unexpected lack of association between *Leucocytozoon* and trypanosome infections in adult sparrowhawks might be due to the occurrence of trypanosomes other than *T. avium*, which are not transmitted by black flies [20]. Another trypanosome species infecting raptors, *T. bennetti*, has recently been shown to be transmitted by biting midges [49]; however, barcoding of trypanosomes occurring in the studied sparrowhawk population revealed that the vast majority of the isolates (96%) belong to *T. avium* s.s., which is transmitted by black flies [36]. It should be noted that, based on indirect evidence, Ashford et al. [13] suggested that *Leucocytozoon* is transmitted to sparrowhawks by biting midges. Recent studies of avian blood parasite life-cycles that include transmission by vectors are scarce; for example, the wide range of avian trypanosome vectors has been revealed only recently (see [37]). In this context, to simply suppose that the distinct raptorial “*Haemoproteus*ˮ lineage is transmitted by biting midges is perhaps not appropriate. The use of similar vectors might explain the positive association of *Haemoproteus* and *Leucocytozoon* infections. Nevertheless, if we follow the conservative presumption that the sparrowhawk *Leucocytozoon* is transmitted by black flies, then the lack of a significant association between *Leucocytozoon* and trypanosomes seems surprising. The proximate mechanism of transmission might influence the apparent discrepancy: *Leucocytozoon* is transmitted by a vector’s bite, inoculating sporozoites with saliva, while *Trypanosoma* is transmitted by ingestion of the vector (not probable in raptors) or via the conjuctiva through prediuresis, a process during which infective stages are expelled with prediuretic liquid while the vector feeds, as was recently demonstrated for *Trypanosoma avium* sensu lato [37]. Consumption of infected prey has been suggested as a mode of transmission for avian trypanosomes in sparrowhawks [50] and since the species is a specialist that feeds almost exclusively on small birds, this additional mode of trypanosome transmission should be considered as well.

At the generic level, *Leucocytozoon* had the highest incidence and persistence of infection. Most individuals acquire their *Leucocytozoon* infection early in life (see preceding text); thus, there is no effect of adult age on prevalence. The incidence of *Trypanosoma* was lower but its persistence was high. Since the incidence does not increase with age, increasing prevalence of trypanosomes with age is probably caused by the accumulation of chronic trypanosome infections. In one study, trypanosome infections caused by the same species (*T. avium* s.s.) were mostly lost in passerines (*Geothlypis trichas*) that were repeatedly sampled [29]. The detection of *T. avium* by culture methods is about twofold more sensitive than by PCR ([37] and Svobodová et al., unpublished), probably due to very low blood parasitemia. Consequently, chronic infections with lower parasitemia might remain undetected, leading to an underestimation of prevalence. Moreover, only seven parasite lineages out of 54 were barcoded in the passerine study; thus, the diversity of the parasites might remain undetected. *Haemoproteus* had the lowest incidence and persistence; nevertheless, *Haemoproteus* prevalence increased with age as well.

The persistence of infection detected at the parasite genus level may in fact hide more or less intensive lineage turnover due to reinfections, since lineages may change while the apparent infection status remains the same. This applies mostly for those parasites with the highest (almost saturated) prevalence (*Leucocytozoon*). Indeed, *Leucocytozoon* sp. lineage changed in one-third of the samples, and species status changed in two-thirds of samples. This result is in concordance with *Leucocytozoon* in the Great Tits (*Parus major*) where lineage turnover was also high; in that study, as many as 17 haplotypes were found in a single population [51]. On the other hand, we found that sparrowhawk individuals that were repeatedly sampled retained their *Haemoproteus* lineages (but the sample size is small).

A high turnover of *Leucocytozoon* lineage further supports the need for parasite barcoding to improve detailed monitoring of intraindividual lineage turnover. On the other hand, the vast majority of trypanosomes found in sparrowhawks belonged to a single *T. avium* lineage, probably not due to host specificity of trypanosomes that belong to other trypanosome lineages but instead due to constraints given by different vectors and transmission modes of those lineages (see preceding text).

## Conclusions

All three genera of blood parasites detected in this study persist in infected individuals, thus enabling sustainability of vector transmission cycles. The prevalence of all three genera of blood parasites increases with age of infected individual; however, a high turnover of *Leucocytozoon* lineages was noted. No clear evidence of parasite-induced mortality was noted, and most of the individuals were found to be infected early in life, particularly in the case of *Leucocytozoon*.

## Supplementary Information

**Additional file 1:** **Table S1.** Logistic regression estimates for predictors of parasite prevalence in adult sparrowhawks, based on all available samples. **Table S2.** Logistic regression estimates for predictors of parasite prevalence in adult sparrowhawks, based on one sample per individual.

**Additional file 2:** **Dataset S1.** Age, sex, year of sampling and infection status of adult sparrowhawks on all sampling occasions.

**Additional file 3: Figure S1.** The effect of infection status on first sampling (0/1) on the prevalence at the next sampling of the same host individual. Marginal means (averaged over sex) with 95% CI, estimated by logistic model with additive effects of initial infection status, sex and age, run separately for each parasite are shown. Sample size for each category is shown above bars (see Table 2). Prevalence of previously uninfected individuals indicates incidence (light shaded bars), prevalence of previously infected individuals indicates persistence (dark shaded bars).

**Additional file 4: Figure S2.** Overall prevalence of *Leucocytozoon* and proportions of different species and lineages. Numbers within bars indicate sample size.

**Additional file 5:** **Figure S3.** Effects of *Leucocytozoon* species and lineages infection status at first sampling (0/1) on the prevalence at the next sampling of the same host individual. Raw proportions with 95% CI, calculated from the data given in Table 3, separately for each *Leucocytozoon *species and lineage are shown. Sample size for each category is shown above bars. Prevalence of previously uninfected individuals (lightly shaded bars) indicates incidence, prevalence of previously infected individuals (dark shaded bars) indicates persistence.

## Data Availability

The sequences obtained in this study were submitted to GenBank and are available under accession numbers ON375833-ON375837. Data for statistical analysis are available in Additional file 2.
