# Supplementary material for: Blood parasites (Trypanosoma, Leucocytozoon, Haemoproteus) in the Eurasian sparrowhawk (Accipiter nisus): diversity, incidence and persistence of infection at the individual level
Source: Parasit Vectors. 2023 Jan 14;16:15. doi: 10.1186/s13071-022-05623-x (PMC9840293; doi:10.1186/s13071-022-05623-x)
Supplement: Supplementary file 1 — Additional file 1: Table S1. Logistic regression estimates for predictors of parasite prevalence in adult sparrowhawks, based on all available samples. Table S2. Logistic regression estimates for predictors of parasite prevalence in adult sparrowhawks, based on one sample per individual. [file 13071_2022_5623_MOESM1_ESM.docx]

**Additional File 1: Table S1**

Logistic regression estimates for predictors of parasite prevalence in adult sparrowhawks, based on all available samples. Model with either linear or quadratic effect of host age was run for a data set with/without two oldest female hosts (aged 9 years), resulting in four models for each parasite. Age was centered before analysis, so that both linear and quadratic terms can be interpreted from the quadratic model. All models included host sex (reference level = female) and year (6 levels) as fixed categorical predictors. An effect of year (results not shown) was not significant in any model but was kept as a blocking variable. Odds ratio (OR = e^Estimate^) is shown with 95% CI. Approximate P-values are based on z-scores of parameter estimates. Sample size indicates number of samples/individuals. Results based on only one sample per individual are shown in Additional File 2: Table S2. Estimated prevalences are shown in Fig. 4

| Response/predictor | Estimate | SE | OR | 95% CI | | P |
| --- | --- | --- | --- | --- | --- | --- |
| *Haemoproteus* (linear effect of host age, 1-9 years, n = 253/190) | | | | | | |
| Age (years) | 0.186 | 0.083 | 1.205 | 1.026 | 1.422 | 0.024 |
| Sex (male) | -1.473 | 0.333 | 0.229 | 0.116 | 0.431 | <0.001 |
| *Haemoproteus* (quadratic effect of host age, 1-9 years, n = 253/190) | | | | | | |
| Age (years) | 0.392 | 0.119 | 1.480 | 1.178 | 1.879 | 0.001 |
| Age^2^ (years) | -0.096 | 0.039 | 0.909 | 0.838 | 0.978 | 0.014 |
| Sex (male) | -1.554 | 0.339 | 0.211 | 0.106 | 0.402 | <0.001 |
| *Haemoproteus* (linear effect of host age, 1-7 years, n = 251/188) | | | | | | |
| Age (years) | 0.252 | 0.088 | 1.287 | 1.085 | 1.536 | 0.004 |
| Sex (male) | -1.500 | 0.335 | 0.223 | 0.112 | 0.421 | <0.001 |
| *Haemoproteus* (quadratic effect of host age, 1-7 years, n = 251/188) | | | | | | |
| Age (years) | 0.371 | 0.124 | 1.449 | 1.142 | 1.857 | 0.003 |
| Age^2^ (years) | -0.067 | 0.048 | 0.936 | 0.852 | 1.027 | 0.162 |
| Sex (male) | -1.541 | 0.338 | 0.214 | 0.107 | 0.407 | <0.001 |
| *Leucocytozoon* (linear effect of host age, 1-9 years, n = 256/193) | | | | | | |
| Age (years) | -0.056 | 0.123 | 0.946 | 0.748 | 1.218 | 0.650 |
| Sex (male) | -0.784 | 0.428 | 0.457 | 0.194 | 1.052 | 0.067 |
| *Leucocytozoon* (quadratic effect of host age, 1-9 years, n = 256/193) | | | | | | |
| Age (years) | 0.079 | 0.162 | 1.082 | 0.787 | 1.492 | 0.627 |
| Age^2^ (years) | -0.061 | 0.048 | 0.941 | 0.856 | 1.039 | 0.205 |
| Sex (male) | -0.830 | 0.434 | 0.436 | 0.182 | 1.015 | 0.056 |
| *Leucocytozoon* (linear effect of host age, 1-7 years, n = 254/191) | | | | | | |
| Age (years) | 0.022 | 0.137 | 1.022 | 0.790 | 1.361 | 0.871 |
| Sex (male) | -0.803 | 0.432 | 0.448 | 0.188 | 1.038 | 0.063 |
| *Leucocytozoon* (quadratic effect of host age, 1-7 years, n = 254/191) | | | | | | |
| Age (years) | 0.046 | 0.172 | 1.047 | 0.744 | 1.470 | 0.790 |
| Age^2^ (years) | -0.016 | 0.071 | 0.984 | 0.860 | 1.141 | 0.823 |
| Sex (male) | -0.812 | 0.434 | 0.444 | 0.185 | 1.033 | 0.062 |
| *Trypanosoma* (linear effect of host age, 1-9 years, n = 209/154) | | | | | | |
| Age (years) | 0.295 | 0.111 | 1.343 | 1.093 | 1.693 | 0.008 |
| Sex (male) | -0.116 | 0.328 | 0.891 | 0.469 | 1.705 | 0.724 |
| *Trypanosoma* (quadratic effect of host age, 1-9 years, n = 209/154) | | | | | | |
| Age (years) | 0.365 | 0.129 | 1.440 | 1.122 | 1.866 | 0.005 |
| Age^2^ (years) | -0.045 | 0.043 | 0.956 | 0.881 | 1.050 | 0.300 |
| Sex (male) | -0.129 | 0.331 | 0.879 | 0.461 | 1.691 | 0.697 |
| *Trypanosoma* (linear effect of host age, 1-7 years, n = 207/152) | | | | | | |
| Age (years) | 0.369 | 0.123 | 1.447 | 1.152 | 1.869 | 0.003 |
| Sex (male) | -0.127 | 0.331 | 0.881 | 0.462 | 1.694 | 0.701 |
| *Trypanosoma* (quadratic effect of host age, 1-7 years, n = 207/152) | | | | | | |
| Age (years) | 0.351 | 0.136 | 1.420 | 1.091 | 1.866 | 0.010 |
| Age^2^ (years) | 0.023 | 0.069 | 1.023 | 0.902 | 1.190 | 0.738 |
| Sex (male) | -0.123 | 0.330 | 0.884 | 0.464 | 1.700 | 0.710 |

**Additional File 1: Table S2**

Logistic regression estimates for predictors of parasite prevalence in adult sparrowhawks, based on one sample per individual. Only the first sample from each host individual was included in these models (n = number of samples = number of individuals). Results based on all available samples are shown in Additional File 1: Table S1

| Response/predictor | Estimate | SE | OR | 95% CI | | P |
| --- | --- | --- | --- | --- | --- | --- |
| *Haemoproteus* (linear effect of host age, 1-9 years, n = 190) | | | | | | |
| Age (years) | 0.146 | 0.104 | 1.157 | 0.943 | 1.426 | 0.162 |
| Sex (male) | -1.288 | 0.371 | 0.276 | 0.129 | 0.558 | 0.001 |
| *Haemoproteus* (quadratic effect of host age, 1-9 years, n = 190) | | | | | | |
| Age (years) | 0.485 | 0.177 | 1.624 | 1.154 | 2.321 | 0.006 |
| Age^2^ (years) | -0.111 | 0.049 | 0.895 | 0.806 | 0.979 | 0.022 |
| Sex (male) | -1.419 | 0.382 | 0.242 | 0.111 | 0.499 | <0.001 |
| *Haemoproteus* (linear effect of host age, 1-7 years, n = 188) | | | | | | |
| Age (years) | 0.259 | 0.118 | 1.295 | 1.031 | 1.640 | 0.028 |
| Sex (male) | -1.356 | 0.376 | 0.258 | 0.120 | 0.526 | <0.001 |
| *Haemoproteus* (quadratic effect of host age, 1-7 years, n = 188) | | | | | | |
| Age (years) | 0.451 | 0.191 | 1.570 | 1.086 | 2.301 | 0.018 |
| Age^2^ (years) | -0.079 | 0.061 | 0.924 | 0.818 | 1.042 | 0.194 |
| Sex (male) | -1.409 | 0.381 | 0.244 | 0.112 | 0.503 | <0.001 |
| *Leucocytozoon* (linear effect of host age, 1-9 years, n = 193) | | | | | | |
| Age (years) | -0.044 | 0.134 | 0.957 | 0.746 | 1.271 | 0.744 |
| Sex (male) | -0.250 | 0.459 | 0.779 | 0.313 | 1.930 | 0.586 |
| *Leucocytozoon* (quadratic effect of host age, 1-9 years, n = 193) | | | | | | |
| Age (years) | 0.080 | 0.218 | 1.084 | 0.709 | 1.680 | 0.713 |
| Age^2^ (years) | -0.039 | 0.053 | 0.962 | 0.866 | 1.074 | 0.467 |
| Sex (male) | -0.300 | 0.466 | 0.741 | 0.293 | 1.859 | 0.521 |
| *Leucocytozoon* (linear effect of host age, 1-7 years, n = 191) | | | | | | |
| Age (years) | 0.055 | 0.163 | 1.057 | 0.784 | 1.501 | 0.734 |
| Sex (male) | -0.303 | 0.465 | 0.738 | 0.293 | 1.846 | 0.514 |
| *Leucocytozoon* (quadratic effect of host age, 1-7 years, n = 191) | | | | | | |
| Age (years) | -0.009 | 0.243 | 0.991 | 0.615 | 1.606 | 0.970 |
| Age^2^ (years) | 0.032 | 0.088 | 1.032 | 0.876 | 1.255 | 0.722 |
| Sex (male) | -0.282 | 0.468 | 0.754 | 0.297 | 1.898 | 0.547 |
| *Trypanosoma* (linear effect of host age, 1-9 years, n = 154) | | | | | | |
| Age (years) | 0.344 | 0.147 | 1.410 | 1.083 | 1.937 | 0.020 |
| Sex (male) | -0.084 | 0.372 | 0.920 | 0.443 | 1.917 | 0.822 |
| *Trypanosoma* (quadratic effect of host age, 1-9 years, n = 154) | | | | | | |
| Age (years) | 0.503 | 0.192 | 1.654 | 1.146 | 2.442 | 0.009 |
| Age^2^ (years) | -0.065 | 0.049 | 0.937 | 0.853 | 1.042 | 0.183 |
| Sex (male) | -0.120 | 0.376 | 0.887 | 0.423 | 1.864 | 0.749 |
| *Trypanosoma* (linear effect of host age, 1-7 years, n = 152) | | | | | | |
| Age (years) | 0.513 | 0.181 | 1.670 | 1.206 | 2.461 | 0.005 |
| Sex (male) | -0.147 | 0.379 | 0.863 | 0.410 | 1.823 | 0.698 |
| *Trypanosoma* (quadratic effect of host age, 1-7 years, n = 152) | | | | | | |
| Age (years) | 0.444 | 0.210 | 1.559 | 1.043 | 2.386 | 0.034 |
| Age^2^ (years) | 0.098 | 0.138 | 1.103 | 0.893 | 1.583 | 0.475 |
| Sex (male) | -0.146 | 0.379 | 0.864 | 0.410 | 1.824 | 0.701 |
