# Supplementary figures and images for: Blood parasites (Trypanosoma, Leucocytozoon, Haemoproteus) in the Eurasian sparrowhawk (Accipiter nisus): diversity, incidence and persistence of infection at the individual level
Source: Parasit Vectors. 2023 Jan 14;16:15. doi: 10.1186/s13071-022-05623-x (PMC9840293; doi:10.1186/s13071-022-05623-x)

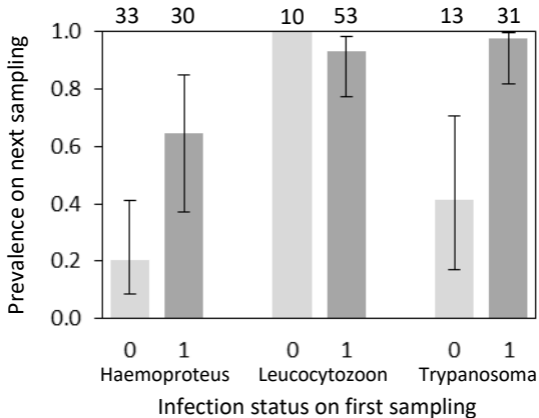

Supplement: Supplementary file 3 — Additional file 3: Figure S1. The effect of infection status on first sampling (0/1) on the prevalence at the next sampling of the same host individual. Marginal means (averaged over sex) with 95% CI, estimated by logistic model with additive effects of initial infection status, sex and age, run separately for each parasite are shown. Sample size for each category is shown above bars (see Table 2). Prevalence of previously uninfected individuals indicates incidence (light shaded bars), prevalence of previously infected individuals indicates persistence (dark shaded bars). [file 13071_2022_5623_MOESM3_ESM.pdf]

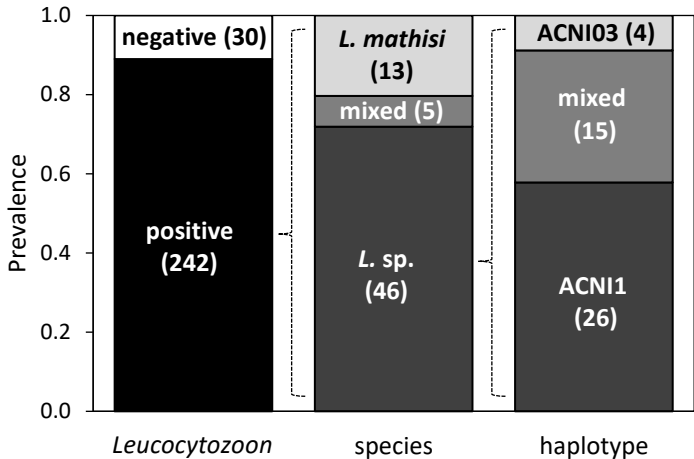

Supplement: Supplementary file 4 — Additional file 4: Figure S2. Overall prevalence of Leucocytozoon and proportions of different species and lineages. Numbers within bars indicate sample size. [file 13071_2022_5623_MOESM4_ESM.pdf]

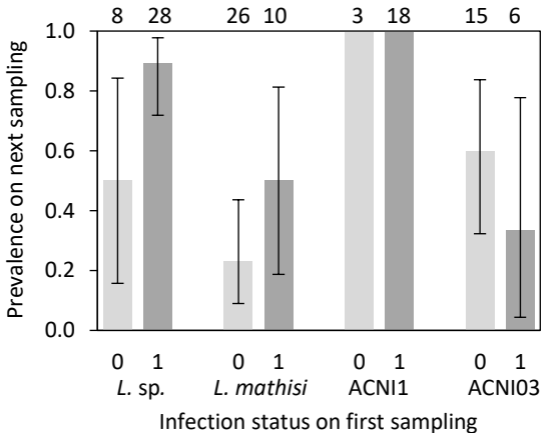

Supplement: Supplementary file 5 — Additional file 5: Figure S3. Effects of Leucocytozoon species and lineages infection status at first sampling (0/1) on the prevalence at the next sampling of the same host individual. Raw proportions with 95% CI, calculated from the data given in Table 3, separately for each Leucocytozoon species and lineage are shown. Sample size for each category is shown above bars. Prevalence of previously uninfected individuals (lightly shaded bars) indicates incidence, prevalence of previously infected individuals (dark shaded bars) indicates persistence. [file 13071_2022_5623_MOESM5_ESM.pdf]
